# Propiedades psicométricas de la versión en español del *Type 2 Diabetes Stigma Assessment Scale* (DSAS 2) en población colombiana

**DOI:** 10.7705/biomedica.6128

**Published:** 2022-05-01

**Authors:** Víctor Pedrero, Luz Marina Alonso, Jorge Manzi

**Affiliations:** 1 Facultad de Enfermería, Universidad Andrés Bello, Santiago, Chile Universidad Andrés Bello Universidad Andrés Bello Santiago Chile; 2 Escuela de Psicología, Pontificia Universidad Católica de Chile, Santiago, Chile Pontificia Universidad Católica de Chile Pontificia Universidad Católica de Chile Santiago Chile; 3 Departamento de Salud Pública, Universidad del Norte, Barranquilla, Colombia Universidad del Norte Universidad del Norte Barranquilla Colombia

**Keywords:** diabetes mellitus, estigma social, reproducibilidad de los resultados., Diabetes mellitus, social stigma, reproducibility of results.

## Abstract

**Introducción.:**

El estigma se ha asociado negativamente al control metabólico y la calidad de vida de pacientes con diabetes de tipo 2. El cuestionario *Diabetes Stigma Assesment Scale* 2 (DSAS 2) fue diseñado para medir específicamente el estigma asociado en personas con este tipo de diabetes. Sin embargo, las propiedades psicométricas de su versión en español aún no han sido analizadas.

**Objetivo.:**

Analizar la validez y confiabilidad de la versión en español del DSAS 2 en población colombiana.

**Materiales y métodos.:**

Se solicitó a 501 pacientes con diabetes de tipo 2 en Barranquilla contestar la versión en español del DSAS 2, un cuestionario con indicadores sociodemográficos y clínicos, así como instrumentos de medición de la depresión, la autoeficacia y el estrés. Se hizo un análisis factorial (exploratorio y confirmatorio) para determinar la estructura interna del DSAS 2 en español y se usó el coeficiente alfa (α) para evaluar su confiabilidad. Además, se analizó la relación entre los puntajes del DSAS 2 y las otras variables estudiadas.

**Resultados.:**

La estructura de tres factores (trato diferente-juicio, culpa y vergüenza) se ajustó adecuadamente a los datos (raíz del error cuadrático medio (RMSEA)=0,081, índice de ajuste comparativo (CIF)=0,959, índice de Tucker-Lewis (TLI)=0,95) y su confiabilidad fue buena (α=0,76). Se observaron correlaciones significativas del puntaje del DSAS 2 en español con la autoeficacia (r_s_=-0,37; p<0,001), el estrés (r_s_=0,24; p<0,001) y la presencia de síntomas depresivos (r_s_=0,1; p=0,021). Además, los puntajes de la escala mostraron variaciones asociadas a variables sociodemográficas y clínicas.

**Conclusiones.:**

La versión en español presenta ciertas diferencias con la versión original del DSAS2, sin embargo, su validez y confiabilidad son suficientes y adecuadas para su uso en Colombia.

## Abstract (en)

**Introduction:**

The perception of stigma has been negatively associated with the metabolic control and quality of life in patients with type 2 diabetes. The Diabetes Stigma Assessment Scale 2 (DSAS 2) was designed to specifically measure the stigma associated with this type of diabetes. However, the psychometric properties of its Spanish version have not yet been addressed.

**Objective:**

To analyze the validity and reliability of the Spanish version of DSAS 2 in a Colombian population.

**Materials and methods:**

In total, 501 patients with type 2 diabetes from Barranquilla answered the Spanish version of DSAS 2, a questionnaire with sociodemographic and clinical indicators, as well as instruments to measure depression, self-efficacy, and stress. We performed a factor analysis (exploratory and confirmatory) to determine the internal structure of the DSAS 2 in Spanish and used the alpha coefficient (a) to evaluate its reliability. Additionally, we analyzed the relationship between the DSAS 2 scores and the other variables under study.

**Results:**

The three-factor structure (different treatment / judgment, guilt and shame) showed good fit to the data (RMSEA = 0.081, CFI = 0.959, TLI = 0.95) and good reliability (α = 0.76). Significant correlations of the scores of the DSAS 2 Spanish version were observed with self-efficacy (r_s_=-0.37, p<0.001), stress (r_s_ =0.24 , p<0.001), and depression (r_s_=0.1, p=0.021). Besides, the scores showed variations associated with sociodemographic and clinical variables.

**Conclusions:**

The Spanish version showed certain differences compared with the original version of the DSAS2 but there was adequate evidence of its validity and reliability to be used in Colombia.

La diabetes de tipo 2 es un importante problema de salud pública a nivel mundial. Actualmente, 463 millones de personas alrededor del mundo tienen este tipo de diabetes y, de estos, aproximadamente 28 millones viven en países latinoamericanos donde el español es el primer idioma ^1^. Específicamente en Colombia, hay cerca de dos millones de personas con esta enfermedad, lo cual equivale a una prevalencia del 8,5 % ^2^^,^^3^. Se espera que en los próximos años estas cifras aumenten debido a factores como el incremento en la esperanza de vida, las tasas de obesidad y el sedentarismo ^4^.

Las cifras de compensación metabólica en la población de Latinoamérica con diabetes de tipo 2 son preocupantes ^4^. Se estima que entre el 3,5 y el 54 % de los pacientes de la región llegarían a niveles de hemoglobina glucosilada menores del 7 % y, en Colombia, solo el 13 % lograría los niveles recomendados ^2^^,^^3^.

Hay acuerdo en que los factores psicosociales, por ejemplo, la autoeficacia, son claves para enfrentar exitosamente la diabetes de tipo 2 ^5^. El estigma hacia la diabetes es un factor psicosocial que recientemente ha despertado interés Internacional debido a su efecto negativo en el manejo y el control de la enfermedad ^5^. Desde un punto de vista conceptual, el estigma es una marca que, dada su connotación social, desacredita a quien la posee ^6^. En este caso, la marca sería poseer la enfermedad y su connotación social respondería a los estereotipos sobre ella, que, en general, enfatizan tanto la responsabilidad individual de las personas por padecerla, como la incapacidad que produce para enfrentar la vida diaria. Estos estereotipos generan un ambiente que propicia el estigma hacia las personas con diabetes ^7^^,^^8^.

El estigma por padecer diabetes opera en diferentes niveles ^9^. Dos de los más abordados son el nivel individual y el interpersonal. El estigma individual (“autoestigma”) alude a la devaluación que las personas con la enfermedad hacen de sí mismas debido a los estereotipos que existen sobre su condición. Por otro lado, el estigma interpersonal ocurre cuando las personas con diabetes de tipo 2 perciben como discriminatorio el trato que reciben de otros (familia, amigos, trabadores de la salud, entre otros).

La percepción del estigma es frecuente en pacientes con diabetes tipo 2 y tiene importantes consecuencias para su salud. En estudios recientes se sugiere que entre el 16 y el 50 % de los pacientes con la enfermedad pueden sentirse estigmatizados ^10^^,^^11^. En este sentido, se ha planteado que la percepción del estigma podría variar según ciertas características de los pacientes como la edad, los años transcurridos desde el diagnóstico o el uso de insulina ^12^^,^^13^. A modo de ejemplo, tener mayor edad se ha asociado con una menor percepción del estigma ^13^.

La evidencia proveniente de Australia, Estados Unidos y Japón sugiere que la percepción de estigma puede afectar negativamente la compensación metabólica y la calidad de vida de las personas con diabetes ^14^^-^^16^. Se ha propuesto que el estrés, la autoeficacia y el desarrollo de síntomas depresivos pueden ser parte de los mecanismos involucrados en estas asociaciones ^9^^,^^14^^-^^17^. Esto significa que parte del efecto del estigma en el control de la diabetes de tipo 2 puede estar mediado por su efecto perjudicial en algunas de tales variables, de ahí la urgencia de avanzar en esta área para contribuir al bienestar de los pacientes.

Actualmente, en Latinoamérica y otras regiones de habla hispana la evidencia sobre este tipo de estigma es prácticamente inexistente. Un factor que contribuye a este vacío es la falta de instrumentos en español para medir el fenómeno. Hoy existen tres instrumentos para evaluar el estigma hacia la diabetes ^9^: dos de ellos se enfocan específicamente en el autoestigma, en tanto que el *Diabetes Stigma Assessment Scale 2* (DSAS 2) es el único que considera tanto el nivel individual como interpersonal del estigma. Este instrumento, creado en Australia, está conformado por tres dimensiones o factores: trato diferente, autoestigma, culpa y juicio. El trato diferente hace referencia a aquellas situaciones en que el paciente ha experimentado discriminación debido a su enfermedad; el autoestigma corresponde al grado en que las personas se aplican a sí mismas los estereotipos sobre la enfermedad, y la escala que mide la culpa y el juicio evidencia hasta qué punto las personas se sienten juzgadas (por su familia y los profesionales de salud, entre otros) y culpables de tener la enfermedad ^11^.

Existe evidencia que respalda la validez y la confiabllidad de la versión en inglés del DSAS 2 ^11^, sin embargo, las propiedades psicométricas de la versión en español aún no han sido analizadas. Dicha evidencia permitiría explorar el problema del estigma en pacientes hispanoparlantes con diabetes de tipo 2, y contribuiría a implementar y evaluar intervenciones desde su perspectiva. Algunas estrategias en este ámbito podrían orientarse a favorecer los mecanismos de afrontamiento al estigma en los pacientes con este tipo de diabetes, así como a fortalecer ciertas habilidades y conocimientos en los trabajadores de la salud ^18^.

El objetivo general del estudio es analizar las evidencias sobre la validez y la confiabilidad de la versión en español del DSAS 2 en población colombiana. Particularmente aborda específicamente los siguientes aspectos: (i) la confiabllidad general de la escala, así como la de cada una de sus subescalas; ii) la estructura interna de esta versión del DSAS 2 evaluada mediante un análisis factorial; (iii) la relación entre los puntajes del instrumento, la presencia de síntomas depresivos, nivel de autoeficacia y el estrés, y (iv) la asociación entre los puntajes del DSAS 2 y las características sociodemográficas y clínicas de la población estudiada.

## Materiales y métodos

### Muestra y procedimiento

Se seleccionó una muestra por conveniencia de 501 participantes en Barranquea, Colombia, durante el 2019. Se incluyeron personas con diagnóstico de diabetes de tipo 2 que tuvieran más de 18 años; no se aplicaron criterios de exclusión. Las encuestas las hizo un encuestador previamente familiarizado con el instrumento y con las condiciones de aplicación (privacidad, prestar asistencia en caso de que los participantes tuvieran dificultades para comprender alguna de las preguntas, y ayuda en la lectura, entre otros aspectos).El cuestionario utilizado incluyó tanto la versión en español del DSAS 2 como otros instrumentos para medir aspectos psicosociales y variables sociodemográficas y clínicas. Antes de ingresar en el estudio, cada participante firmó un consentimiento informado previamente aprobado por el comité de ética de la Universidad del Norte en Colombia (acta de evaluación n^o^ 197).

### Instrumentos

Se utilizó una versión en español del DSAS 2 provista por *Mapi Research Trust*, institución internacional especialista en resultados reportados por pacientes (*Patient Reported Outcomes*) y encargada de la distribución de las versiones del DSAS 2 (acuerdo N^o^ 211063). Dicha versión fue revisada por el equipo de investigación para detectar términos que pudieran ser poco claros o no aplicables a la población de interés, y no se encontraron elementos que debieran modificarse.

El DSAS 2 tiene 19 ítems con una escala de respuesta del tipo Likert de cinco puntos, que van desde “completamente de acuerdo” hasta “completamente en desacuerdo’.’ Estos ítems se distribuyen en tres subescalas: trato diferente, culpa y juicio, y autoestigma. La escala de trato diferente, cuenta con seis ítems; la subescala de culpa y ser objeto de juicio, tiene siete ítems; y la subescala de autoestigma, incluye seis ítems. El puntaje del DSAS 2 oscila entre 19 y 95 puntos, cuanto mayor sea este, mayor es el nivel de estigma. La confiabilidad de la escala completa en el instrumento original, evaluada mediante el alfa de Cronbach (α), es de 0,95; la de trato diferente es de 0,88; la de juicio y culpa, de 0,8, y la de autoestigma, de 0,9 ^11^.

*Autoeficacia.* Los pacientes contestaron los siete ítems de esta escala desarrollada en español por Lorig, *et al*. ^19^ la cual ha sido recomendada previamente para su uso en pacientes con diabetes ^20^. La escala mide la percepción que las personas con diabetes de tipo 2 tienen de su capacidad para lidiar con la enfermedad. Un ejemplo de los ítems presentes en esta escala es: ¿Qué tan seguro(a) se siente usted de poder comer sus alimentos cada 4 o cinco horas todos los días?. La respuesta a cada ítem se puntúa de 1 a 10, siendo los mayores puntajes indicativos de un mayor nivel de autoeficacia. La confiabilidad de esta escala en nuestro estudio fue de α=0,81.

*Síntomas depresivos*. Para su evaluación se utilizó la versión en español *del Personal Health Questionnaire* 2 (PHQ-2) ^21^, el cual incluye dos ítems (En las dos últimas semanas, ¿qué tan seguido ha tenido molestias debido a los siguientes problemas: poco interés o placer en hacer cosas? y ¿Se ha sentido decaído, deprimido o sin esperanzas?), con cuatro alternativas de respuesta (entre “ningún día” y “casi todos los días”). Se ha comprobado que esta es una herramienta válida y confiable para el tamizaje de la depresión. La confiabilidad del instrumento en la muestra estudiada fue de α=0,81.

*Estrés psicológico asociado con la diabetes.* Los participantes completaron la versión en español del instrumento *Problem Areas in Diabetes (PAID)*-5, ya empleado en investigaciones anteriores ^22^^-^^24^. Este instrumento mide el grado de estrés psicológico que enfrentan los pacientes con diabetes por padecerla. Un ejemplo de ítem presente en esta escala es: ¿Cuál de los siguientes aspectos son actualmente un problema para usted?. El tener que lidiar con las complicaciones de la enfermedad. Cada uno de los cinco ítems se responde con una escala del tipo Likert de cinco puntos (entre “no es un problema” y “es un problema muy serio”). Los puntajes más altos indican mayor estrés psicológico. La confiabilidad del instrumento en nuestro estudio fue de α=0,75.

### Variables sociodemográficas y cibicas

Las variables sociodemográficas y las clínicas se seleccionaron por haberse asociado con el estigma en estudios previos ^13^^,^^16^^,^^25^. Las sociodemográficas incluyeron el sexo del participante, su edad (en los rangos que figuran en el *Medical Subject Headings* - MeSH) ^26^ y nivel educativo (primaria, secundaria o superior), así como su régimen de afiliación al Sistema General de Seguridad Social, diferenciando entre los afiliados al régimen contributivo y aquellos sin capacidad de pago pertenecientes al subsidiado ^27^. Entre las variables clínicas, se incluyeron los años transcurridos desde el diagnóstico de diabetes de tipo 2 (<5 años; 5-15 años, >15 años), el uso de insulina (sí/no) y el reporte de la propia persona de la presencia o ausencia de cualquier complicación asociada con la enfermedad (problemas renales, oculares, heridas o pérdida de extremidades). Además, para una aproximación al cumplimiento del tratamiento, se utilizó el número de controles médicos relacionados con la diabetes a los cuales el paciente reportó haber asistido en el último año (cuadro 1).

**Cuadro 1:** Caracterización de la muestra

		N	%
Sexo	Hombre	184	36,7
	Mujer	317	63,3
Edad	18-44	42	8,4
	45-64	265	52,9
	65-80	168	38,5
	>80	26	19
Educación	Primaria	213	42,5
	Secundaria	193	38,5
	Superior	95	19
Régimen de afiliación al	Contributivo	175	35
sistema de salud	Subsidiado	325	65
Años con diabetes	≤5	225	44,9
	6-15	192	38,3
	>15	84	16,8
Uso de insulina	No	323	64,5
	Sí	178	35,5
Complicaciones asociadas	No	368	73,9
a la diabetes^§^	Sí	130	26,1
Controles durante el último año (Mdn)^§§^		501	4

^§^ Reporte del propio paciente sobre la presencia de alguna de las siguientes complicaciones: problemas renales, oculares, heridas o pérdida de extremidades

^§^ ^§^ En el caso de esta la variable, se reporta como valor estadístico descriptivo la mediana (Mdn).

### Análisis de la estmctura factorial

El análisis factorial confirmatorio (AFC) y el exploratorio (AFE) sirvieron para evaluar la estructura factorial de la versión en español del DSAS 2. En ambos casos, y dada la naturaleza ordinal de su escala de respuesta, las estimaciones se hicieron a partir de la matriz de correlaciones de los ítems del cuestionario. Tres índices de bondad de ajuste permitieron evaluar el ajuste global de cada modelo a los datos: la raíz del error cuadrático medio (RMSEA), el índice de ajuste comparativo (CFI) y el índice de Tucker-Lewls (TLI) (28). Se consideró que el modelo se ajustaba bien si el CFI y el TLI eran mayores o ¡guales a 0,9 y la RMSEA menor o igual a 0,08 ^28^.

Específicamente en el caso del AFE, se estimó el coeficiente de Kaiser- Meyer-Olkin (KMO) para evaluar la pertinencia de realizar el análisis (los valores de KMO superiores a 0,7 indican que los datos resultan adecuados para el análisis). La solución factorial se rotó de manera oblicua (Geomln), ya que los autores de esta escala señalan que existe correlación entre sus diferentes factores. La decisión sobre el número de factores por extraer se basó en las siguientes consideraciones: i) el criterio de Kaiser (*Eigen value* ≥ 1); ii) el gráfico de sedimentación; ¡ii) el porcentaje de varianza explicada; iv) el sentido teórico de la solución factorial, y v) las cargas factoriales (≥ 0,3). Las estimaciones para ambos análisis (AFC y AFE) se hicieron en el programa Mplus 8.

Para determinar la confiabilidad total y la de cada subescala, se empleó el coeficiente alfa de Cronbach (α). Los patrones de respuesta y los puntajes finales se analizaron mediante estadística descriptiva.

### Relación con otras variables

Otra evidencia de validez surge del grado en que el DSAS 2 se relaciona con otras variables según lo esperado. En este sentido, se estimó la correlación entre el puntaje obtenido en la versión en español del DSAS 2 (total y para cada una de sus subescalas) y los puntajes de autoeficacia, estrés y presencia de síntomas depresivos. Se esperaba que los puntajes del DSAS 2 se correlacionaran positivamente con el estrés y la presencia de síntomas depresivos, y negativamente, con la autoeficacia. Esto sugiere que, a mayor percepción del estigma, mayor estrés, mayor sintomatologia depresiva y menores niveles de autoeficacia, hipótesis que concuerda con lo planteado previamente ^14^^-^^16^. Dada la falta de normalidad de la versión en español del DSAS 2, comprobada mediante la prueba de Kolmogorov- Smirnov (D=0,065; p<0,001), para estos análisis se utilizó la correlación de Spearman (r_s_). Se aceptó como significativo un valor de p<0,05.

Por último, se exploró la asociación entre los puntajes del DSAS 2 en español, y las variables sociodemográficas y clínicas, para lo cual se empleó la prueba U de Mann-Whitney cuando se trataba de dos grupos y, la de Kruskall Wallis, cuando eran más de dos grupos (educación y años transcurridos desde el diagnóstico de diabetes de tipo 2). Cuando se trataba de dos grupos (sexo), se aceptó como significativo un valor de p<0,05 y, cuando el número de grupos era mayor, se aplicó el ajuste de Bonferroni para comparaciones múltiples. Como producto de este ajuste, se aceptó un nivel de significación de p<0,017 para la asociación entre el nivel de estigma y la edad, y uno de p<0,008, para la asociación entre el nivel de estigma, el nivel educacional y los años transcurridos desde el diagnóstico de la enfermedad. Estas estimaciones se hicieron en el programa SPSS, versión 25.

## Resultados

El análisis descriptivo de los patrones de repuesta evidenció una tendencia de los participantes a preferir los extremos de la escala (cuadro 2). En 11 de los 19 ítems una proporción igual o superior al 80 % de los participantes prefirió los extremos, positivos o negativos, de la escala de respuesta, en tanto que, en promedio, solo 0,7 % seleccionó la opción intermedia (‘no estoy seguro’) (cuadro 2).

**Cuadro 2:** Estadísticos descriptivos para cada uno de los ítems de la versión en español del DSAS 2

	Estoy completamente en desacuerdo		Estoy en desacuerdo		No estoy seguro		Estoy de acuerdo		Estoy completamente de acuerdo.		M	DE
	N	%	N	%	N	%	N	%	N	%		
Algunas personas piensan que no puedo cumplir con mis responsabilidades (por ejemplo: trabajo, familia) porque tengo diabetes tipo 2.	32	6,4	302	60,3	1	0,2	160	31,9	6	1,2	2,61	1,04
Porque tengo diabetes tipo 2, algunos profesionales de la salud	18	3,6	178	35,5	0	0,0	268	53,5	37	7,4	3,26	1,13
me han juzgado de manera negativa. Porque tengo diabetes tipo 2, algunas personas suponen que tengo sobrepeso o que he tenido sobrepeso en el pasado.	12	2,4	193	38,6	1	0,2	201	40,2	93	18,6	3,34	1,23
Algunas personas me tratan como si estuviera “enfermo/a” porque tengo diabetes tipo 2.	35	7,0	226	45,1	0	0,0	230	45,9	10	2,0	2,91	1,12
Hay culpa y vergüenza con la diabetes tipo 2.	19	3,8	462	92,2	1	0,2	19	3,8	0	0,0	2,04	0,44
Me siento avergonzado/a por mi diabetes tipo 2	21	4,2	455	90,8	2	0,4	22	4,4	1	0,2	2,06	0,49
Me han discriminado en el trabajo por tener diabetes tipo 2.	31	6,2	431	86,2	2	0,4	34	6,8	2	0,4	2,09	0,61
Algunos profesionales de la salud piensan que las personas con diabetes tipo 2 no saben cuidarse.	17	3,4	40	8,0	0	0,0	303	60,8	138	27,7	4,01	0,95
Me da vergüenza tener diabetes tipo 2.	57	11,4	421	84,0	2	0,4	21	4,2	0	0,0	1,97	0,53
Algunas personas me ven como una persona inferior porque tengo diabetes tipo 2.	360	71,9	131	26,1	3	0,6	7	1,4	0	0,0	1,32	0,56
Porque tengo diabetes tipo 2, siento que no soy lo suficientemente bueno/a.	391	78,0	104	20,8	1	0,2	5	1,0	0	0,0	1,24	0,50
La diabetes tipo 2 tiene un estigma negativo por ser una enfermedad de ‘estilo de vida’.	8	1,6	53	10,6	21	4,2	382	76,4	36	7,2	3,77	0,79
Tener diabetes tipo 2 me hace sentir como un fracaso.	170	33,9	330	65,9	1	0,2	0	0,0	0	0,0	1,66	0,48
Algunas personas me excluyen de eventos sociales donde hay comidas o bebidas que piensan que no debo comer o beber.	24	4,8	337	67,5	2	0,4	117	23,4	19	3,8	2,54	1,02
Me siento culpable por tener diabetes tipo 2.	17	3,4	274	54,8	6	1,2	196	39,2	7	1,4	2,80	1,05
Me han dicho que yo he causado mi diabetes tipo 2.	11	2,2	166	33,3	2	0,4	231	46,3	89	17,8	3,44	1,18
He sido rechazado/a por otras personas (por ejemplo: amigos, colegas, parejas) porque tengo diabetes tipo 2.	16	3,2	460	91,8	0	0,0	24	4,8	1	0,2	2,07	0,49
Me culpo a mí mismo/a por tener diabetes tipo 2.	17	3,4	244	48,8	14	2,8	219	43,8	6	1,2	2,91	1,05
Porque tengo diabetes tipo 2, algunas personas me juzgan por las comidas que escojo.	6	1,2	91	18,2	3	0,6	308	61,5	93	18,6	3,78	0,99

M: media, DE: desviación estándar. ítems que conforman cada subescala en la versión en inglés. Trato diferente: 1,4,7,10,14,17. Culpa y ser juzgado: 2,3,5,8,12,16. Autoestigma: 6,9,11,13,15,18

Después del análisis descriptivo, se sometió a prueba la estructura factorial original del DSAS 2 utilizando un AFC, el cual no mostró un buen ajuste a los datos (RMSEA=0,142; CFI=0,758). Por esta razón, se optó por hacer un análisis factorial exploratorio. Un resultado en el índice de Kayser- Meyer-Olkin de 0,74,indicó que la muestra era adecuada para la realización del AFE. El análisis del gráfico de sedimentación evidenció que una solución de cuatro factores sería adecuada. Si bien el quinto factor también poseía un *Eigenvalue* mayor de 1 (1,096), este no agrupó ítems cuya interpretación tuviera sentido teórico. La solución de cuatro factores explicó el 61 % de la varianza presente en los datos y mostró un buen ajuste (RMSEA=0,069; CFI=0,97; TLI=0,95). Las cargas factoriales oscilaron entre 0,43 y 0,93 (cuadro 3). El análisis de confiabilidad mostró valores por encima de 0,7 para tres de los cuatro factores, en tanto que la confiabilidad del factor restante fue extremadamente baja (α=0,25).

**Cuadro 3:** Análisis factorial para la escala DSAS 2 en español

		Análisis factorial exploratorio				Análisis factorial confirmatorio			Análisis factorial confirmatorio		
		F1	F2	F3	F4	F1: juicio/trato diferente	F2: Culpa	F3: Vergüenza	F1: juicio/trato diferente	F2: Culpa	F3: Verguenza
ítems	1	0,847				0,728			0,73		
	2	0,584				0,639			0,641		
	3	0,312				0,395			0,399		
	4	0,742				0,742			0,74		
	5				0,716			0,841			0,841
	6				0,826			0,908			0,908
	7	0,419				0,603			0,597		
	8	0,521				0,563			0,566		
	9				0,933			0,819			0,811
	10		0,771				-	-	-	-	-
	11		0,732				-	-	-	-	-
	12		0,523				-	-	-	-	-
	13				0,431			0,272	-	-	-
	14	0,447				0,517			0,519		
	15			0,93			0,895			0,895	
	16			0,696			0,719			0,721	
	17	0,645				0,717			0,711		
	18			0,938			0,97			0,97	
	19	0,443				0,494			0,494		
Correlación entre factores	F2: Culpa					0,325			0,326		
	F3: Vergüenza					0,525	0,38		0,535	0,386	
Índices de ajuste	RMSEA		0,069				0,08			0,081	
	CFI		0,97				0,955			0,959	
	TLI		0,95				0,947			0,95	
	Alfa	0,71	0,254	0,86	0,73	0,709	0,86	0,728	0,709	0,86	0,855

Dado que los resultados del AFE sugieren que una estructura de tres factores se ajustaría más a los datos, se empleó un AFC para validar dicha estructura considerando 16 ítems después de eliminar los tres que mostraron problemas en el análisis previo. El modelo con tres factores tuvo un buen ajuste a los datos (RMSEA=0,08; CFI=0,955; TLI=0,947). Sin embargo, el ítem 13 presentó una carga factorial de 0,27, por lo que se eliminó y el modelo confirmatorio se estimó nuevamente (cuadro 3). El modelo final mostró un buen ajuste (RMSEA=0,081; CFI=0,959; TLI=0,95). La correlación entre factores varió entre r=0,33 (p<0,05) y r=0,54 (p<0,05), lo cual indica que cada subescala refleja un aspecto distinto del estigma hacia la diabetes, diferenciándose de las otras (cuadro 3).

Los 15 ítems que conformaron el DSAS 2 en el modelo final se distribuyeron de la siguiente manera: una subescala con nueve ítems que aborda conjuntamente la percepción de juicio y el trato diferente; una con tres ítems que aborda la culpa asociada a la enfermedad, y una tercera también compuesta por tres ítems que aborda los sentimientos de vergüenza asociados con tener diabetes. La confiabilidad de la escala completa fue de α=0,76, la de la primera subescala, de α=0,71, y la de las otras dos, de α=0,86. Vale la pena señalar que la confiabildiad de la escala con 19 ítems fue muy similar a la de la escala con 15 ítems. Además, la correlación entre ambas fue muy alta.

La media de los puntajes obtenidos para la escala de 15 ítems fue de 41,8 y la desviación estándar (DE) fue de 6,7, con una mediana de 41 y un rango que osciló entre los 18 y 64 puntos. En el caso de las subescalas, las medias y desviaciones estándar fueron: juicio/trato diferente, media=25,58 y DE=4,83 (mediana=26; rango=12-41); culpa, media=9,15 y DE=2,9 (mediana=8; rango 3-14); vergüenza, media=6,07 y DE=1,29 (mediana=6; rango 3-13).

Se observaron correlaciones significativas entre el puntaje global para el estigma y la autoeficacia (r_s_=-0,37; p<0,001), el estrés (r_s_=0,24; p<0,001), y la presencia de síntomas depresivos (r_s_=0,1; p=0,021) (cuadro 4). El mismo patrón se observó al analizar la correlación entre la subescala de trato diferente/juicio y estas variables. Las subescalas de culpa y vergüenza solo se correlacionaron significativamente con la autoeficacia (r_s_=-0,18; p<0,001; r_s_=-0,1; p=0,031, respectivamente) (cuadro 4).

**Cuadro 4.** Correlaciones entre la versión en español del DSAS 2 y las variables de interés

	1	2	3	4	5	6	7
DSAS 2	1						
DSAS 2: Trato diferente/juicio	0,86**	1					
DSAS 2: Culpa	0,64**	0,22**	1				
DSAS 2: Vergüenza	0,29**	0,2**	0,1**	1			
Estrés	0,24**	0,27**	0,05	0,04	1		
Autoeficacia	-0,37**	-0,39**	-0,18**	-0,1*	-0,24**	1	
Síntomas depresivos	0,1*	0,14**	-0,05	0,01	0,35**	-0,06	1

p<0,01**; *p<0,05. DSAS: *Diabetes Stigma Assessment Scale*

Por último, se exploró la asociación entre las variables sociodemográficas y clínicas, y los puntajes del instrumento final (cuadro 5). El ser mujer, el tener menos edad y un menor nivel educativo, el pertenecer al sistema subsidiado de salud, el haber sido diagnosticado más recientemente, el no usar insulina y no presentar complicaciones derivadas de la enfermedad, se asociaron con una mayor percepción del estigma. De las tres subescalas de la versión en español del DSAS 2, la de culpa evidenció la relación más constante con estas variables. Sus puntajes también se asociaron significativamente con el número de controles a los cuales el paciente manifestó haber asistido durante el año anterior. La percepción de ser tratado de forma diferente solo se asoció con el tipo de afiliación al sistema de salud. La subescala de vergüenza no mostró relación con las variables estudiadas (cuadro 5).

**Cuadro 5:** Asociación de las diferentes variables sociodemográficas y clínicas con los puntajes del DSAS 2 en español y con cada una de sus subescalas

		DSAS2 (Mdn)	p	Juicio/ trato diferente	p	Culpa (Mdn)	p	Vergüenza (Mdn)	p
Sexo	Hombre	40	0,015	26	0,333	8	0,002	6	0,274
	Mujer	42		26		10		6	
Edad*	18-44	40	0,001	26	0,04	8	0,001	6	0,809
	45-64	42		26		10		6	
	65-80	41		26		8		6	
	>80	38		24,5		8		6	
Educación*	Primaria	42	0,001	27	0,06	10	<0,001	6	0,965
	Secundaria	41		26		9,5		6	
	Superior	40		25		7		6	
Régimen de afiliación al	Contributivo	40	<0,001	26	0,001	8	0,002	6	0,961
sistema de salud	Subsidiado	42		27		10		6	
Años con* diabetes	≤5	43	<0,001	26	0,95	12	<0,001	6	0,568
	6-15	40		26		8		6	
	>15	39		26,5		6		6	
Uso de insulina	No	42	0,017	26	0,79	10	<0,001	6	0,607
	Sí	40		26		8		6	
Complicaciones	No	42	0,975	26	0,248	9	0,088	6	0,846
asociadas a la diabetes^§^	Sí	41		27		8		6	
Controles durante el último año^§§^		-0,05	0,239	-0,004	0,923	-0,14	0,002	0,04	0,351

*Debido a las comparaciones múltiples se consideró una corrección de Bonferroni. Según esta corrección para la educación, se aceptó como significativo un p<0,017, en tanto que para la edad y los años con diabetes se aceptó como significativo un p<0,008.

^§^Reporte de la propia persona sobre la presencia de alguna de las siguientes complicaciones: problemas renales, oculares, heridas o pérdida de extremidades

^§§^ En el caso de esta la variable, se tomó como medida de asociación la correlación de Spearman considerando significativo un p<0,05. Mdn: mediana

## Discusión

El análisis de la versión en español del DSAS 2 mostró que la inclusión de tres factores y 15 ítems era apropiada. Esta versión evidenció buenos índices de confiablidad global y de cada una de las tres subescalas. Los puntajes obtenidos se correlacionaron según lo esperado con los niveles de autoestigma y estrés, y con la presencia de síntomas depresivos en la muestra analizada. Hubo una asociación significativa tanto de los puntajes de la escala global como de la subescala de culpa con las características sociodemográficas y clínicas, lo que no se observó con la subescala de vergüenza.

Actualmente, hay dos versiones del DSAS 2 que han mostrado evidencias de su validez y confiabilidad: la original, desarrollada en Australia ^11^, y una adaptación reciente hecha en Turquía ^29^, ambas con la misma estructura de tres factores. La versión en español de DSAS 2 también posee tres factores, pero con variaciones en la forma en que se distribuyen los ítems. En el presente estudio, la subescala de trato diferente/juicio (9 ítems) incluyó cinco de los seis ítems originales de la subescala de trato diferente, y cuatro de los siete de la de culpa y juicio. Los nueve ítems resultantes abordan tanto la percepción de sentirse discriminado (trato diferente) como la de sentirse enjuiciado por otros (prejuicio). Un elemento que puede explicar estos resultados es la estrecha relación existente entre estigma y prejuicio. Después de analizar diferentes modelos teóricos, Phelan, *et al*. ^30^, sugirieron que hay importantes similitudes entre prejuicio y estigma, especialmente, cuando se trata de su faceta interpersonal, y que una de sus principales diferencias radicaría en el tipo de característica asociada con estos constructos analizada en cada investigación: en los estudios centrados en el prejuicio, se han abordado con mayor frecuencia aspectos como el origen racial y, en los orientados al estigma, la enfermedad y la discapacidad, lo que ayudaría a explicar la configuración que adoptó esta escala en nuestro estudio.

Los ítems de dos de las subescalas originales del DSAS 2 (autoestigma y culpa/juicio) se configuraron en dos nuevas subescalas: una que da cuenta de los sentimientos de culpa derivados de sufrir la enfermedad y otra que representa la percepción de vergüenza asociada con la diabetes. Originalmente, cuatro de los seis ítems de estas dos nuevas subescalas pertenecían a la subescala de autoestigma. Corrigan, *et al*. ^31^, definieron autoestigma como el proceso mediante el cual las personas adoptan para sí mismas los estereotipos que existen sobre su condición, en este caso, aquellos asociados con tener diabetes de tipo 2. Según los autores, al apropiarse de tales estereotipos, las personas se enjuician y terminan teniendo una percepción devaluada de sí mismas. Tanto la culpa como la vergüenza pueden aparecer como consecuencia de este juicio personal. Estas dos emociones se correlacionan entre sí, tal como se apreció en nuestro estudio, pero presentan importantes diferencias ^32^^,^^33^. La vergüenza implica una devaluación completa de la propia imagen debido a una determinada condición (por ejemplo, tener diabetes), en tanto que la culpa se produce como consecuencia de un acto específico que se considera negativo y sobre el cual tenemos control. Esta diferencia sugiere que, en el caso de la diabetes, ambas emociones podrían derivarse de estereotipos distintos sobre la enfermedad: los que aluden a la diabetes como una enfermedad incapacitante podrían conducir a un daño global de la autoimagen e inducir vergüenza, y aquellos que aluden a la responsabilidad individual de las personas por poseer la enfermedad podrían precipitar la aparición de la culpa ^7^^,^^8^.

De los 19 ítems de la escala original, cuatro se eliminaron en la versión en español. Tres de ellos, con los puntajes más bajos en la muestra analizada, se refieren a sentimientos de inferioridad y fracaso personal que, si bien podrían compartir ciertas similitudes con emociones como la vergüenza, al parecer responden a concepciones distintas de la enfermedad. Es decir, estereotipos no necesariamente ligados a la aparición de la vergüenza precipitarían este tipo de sentimientos de inferioridad. Un cuarto ítem que no mostró buen comportamiento fue el 12, que, a diferencia de los demás, hace referencia a una característica general del estigma hacia la diabetes, pero no alude específicamente a la experiencia personal, lo que explicaría que no apareciera asociado con las dimensiones establecidas.

En su conjunto, la escala mostró asociaciones significativas con la autoeficacia, el estrés y la presencia de síntomas depresivos, lo que concuerda con lo reportado previamente ^14^^-^^16^. Este mismo patrón se observó en la escala de trato diferente y juicio. Sin embargo, las de vergüenza y culpa solo se relacionaron con la autoeficacia, lo que daría luces sobre las diferencias en los mecanismos involucrados con el estigma en pacientes con diabetes de tipo 2. Previamente, se ha reportado que el autoestigma, constructo relacionado con la culpa y la vergüenza, afectaría principalmente la autoeficacia de los pacientes, perjudicando su capacidad para involucrarse en aspectos importantes de su propio manejo de la enfermedad ^17^^,^^31^. Por otro lado, Dawson, *et al*. ^34^, sugieren que el estrés sería una variable mediadora en la relación entre la percepción de discriminación y la compensación de pacientes con diabetes.

En cuanto a la asociación de los puntajes del DSAS 2 con las variables sociodemográficas y clínicas, un diagnóstico más reciente de la enfermedad se asoció con una mayor percepción del estigma, tal como se ha demostrado previamente. Algunos autores han sugerido que, cuanto mayor es el tiempo en que una persona vive con una característica estigmatizada, mejores son sus mecanismos de afrontamiento ^35^^,^^36^, lo que ayudaría a explicar por qué las personas de menos edad, que no usan insulina ni presentan complicaciones, perciben más el estigma. Al analizar algunas variables vinculadas con el nivel socioeconómico de los participantes (nivel educativo y tipo de régimen de salud), se observó que niveles socioeconómicos más bajos se relacionaron con mayores niveles de estigma. Estos grupos serían más sensibles a las situaciones que implican estigma y discriminación porque usualmente experimentan la intersección de varias condiciones de este tipo ^25^. En cuanto al cumplimiento de los controles médicos, hubo una asociación negativa entre esta variable y la percepción del estigma, lo que concuerda con el hallazgo previo de que mayores niveles de estigma se asociarían con un menor uso de los servicios de salud ^37^. La asociación de los puntajes del DSAS 2 con variables psicosociales, sociodemográficas y clínicas reforzó la validez del instrumento.

Por último, es relevante analizar los patrones de repuesta en el DSAS 2, los cuales pueden verse influenciados por elementos culturales que afectan la comparabilidad del instrumento según el contexto (por ejemplo, diferentes países) ^38^^-^^40^. En el presente estudio, se observó una tendencia de los participantes a preferir los extremos de la escala (positivos o negativos), estilo de repuesta que se conoce como “extremo”. Estos hallazgos difieren de los reportados en el estudio original del DSAS 2 en población australiana ^11^, el único con evidencia detallada sobre el comportamiento de cada ítem hasta la fecha. En dicho estudio se observó que, aunque muchas preguntas presentaban distribuciones con asimetría hacia la derecha, los pacientes utilizaban con mayor frecuencia la escala de respuesta completa. En diversos estudios se ha evidenciado la influencia cultural en la forma en que las personas enfrentan las escalas de tipo Likert ^38^^-^^40^, especialmente cuando hay un alto grado de distancia con el poder, algo más frecuente en países latinoamericanos que en Australia, un aspecto que se relaciona relaciona con la tendencia de las personas a responder en los extremos de la escala ^39^.

Los hallazgos de este estudio tienen implicaciones para la investigación y la práctica. En la primera, la herramienta permitirá profundizar en los mecanismos del estigma hacia la diabetes y conocer más detalladamente los factores asociados en Colombia y otros países de habla hispana. Por otro lado, sería útil en el ámbito clínico para conocer el grado en que las personas con diabetes de tipo 2 se sienten estigmatizadas e intervenir en este sentido para mejorar su bienestar y prevenir complicaciones derivadas de la enfermedad. En este sentido, los enfoques que consideren tanto a los pacientes como a los proveedores de salud podrían ser efectivos ^18^. Por ejemplo, las intervenciones grupales en estos pacientes podrían disminuir la percepción de discriminación e incrementar la autoeficacia ^41^. Nuestros resultados también sugieren que ciertos grupos, como los integrados por aquellas personas diagnosticadas más recientemente, podrían beneficiarse en mayor medida con estas intervenciones. Otras estrategias podrían involucrar el desarrollo de ciertas habilidades en los profesionales de salud que fortalezcan la relación terapéutica y ayuden a los pacientes a combatir el estigma ^14^.

Aunque el estudio tiene alcances relevantes, hay algunos aspectos que deben considerarse al interpretar los resultados. Dada la importancia de los estereotipos asociados con la estigmatización de la diabetes, es relevante explorar cómo se expresan estos en países como Colombia, lo que contribuiría a comprender las variaciones observadas en la configuración de los factores del DSAS 2. Además, se recomienda recoger otras evidencias de validez que exploren detalladamente los procesos de repuesta de los participantes, es decir, el grado en el que los ítems de un instrumento activan los procesos mentales esperados ^42^.

La versión en español del DSAS 2 mostró ciertas variaciones con respecto a la versión original en inglés, sin embargo, demostró suficiente validez y confiabilidad para su uso en población colombiana. Esta escala constituye una herramienta útil en el contexto clínico y en la investigación para abordar el problema del estigma en pacientes con diabetes de tipo 2.
